# CpG Methylation in the Hexamerin 110 Gene in the European Honeybee, *Apis mellifera*

**DOI:** 10.1673/031.011.7401

**Published:** 2011-06-29

**Authors:** Takashi Ikeda, Seiichi Furukawa, Jun Nakamura, Masami Sasaki, Tetsuhiko Sasaki

**Affiliations:** ^1^ Brain Science Institute, Tamagawa University, Tamagawagakuen, Machida, Tokyo 194–8610.; ^2^ Honeybee Science Research Center, Research Institute, Tamagawa University, Tamagawagakuen, Machida, Tokyo 194–8610; ^3^ Section of Molecular Biology and Cell Engineering, Department of Regenerative Medicine, Research Institute, National Center for Global Health and Medicine, Shinjuku, Tokyo 162–8655; ^4^ Biosphere Resource Science and Technology, Graduate School of Life and Environmental Sciences, University of Tsukuba, Tennodai, Tsukuba, lbaraki 305–8572

**Keywords:** bisulfite sequencing, caste differentiation, epigenetics, Hex 110

## Abstract

The European honeybee, *Apis mellifera* L. (Hymenoptera: Apidae), has a full set of machinery for functional CpG methylation of its genome. A recent study demonstrated that DNA methylation in the honeybee is involved in caste differentiation. In this study, the expression and methylation of the hexamerin 110 gene (*Hex110*), which encodes a storage protein, was analyzed. High levels of the *Hex110* transcript were expressed in both worker and queen larvae. Low levels of this transcript were also detected in adult fat bodies, and the expression level was higher in the queen than in the worker. Bisulfite sequencing revealed that the *Hex110* gene is overall methylated at a low level, with a limited number of CpG sites methylated at relatively high levels. These highly methylated sites were exclusively located in the exon regions. The average methylation rate of the *Hex110* gene was higher in the adult stage than in the larval stage. Furthermore, several CpG sites were differentially methylated between the worker and queen larvae. These observations suggest that the methylation of the *Hex110* gene is regulated at the developmental stage and in a caste-dependent manner.

## Introduction

A honeybee colony consists of a queen, many nonreproductive females (workers), and male bees (drones). The mechanism of sex determination in honeybees is a haplodiploid system in which the males develop from unfertilized eggs and the females develop from fertilized eggs. Differentiation of a female larva into a particular caste is not determined genetically, but by environmental factors. A larva hatched in a special cell called a queen cell is fed with only royal jelly, and differentiates into a queen. Larvae hatched in ordinary bee cells are fed with worker jelly containing pollens and develop into workers.

The European honeybee, *Apis mellifera* L. (Hymenoptera: Apidae), has the de novo cytosine methyltransferase Dnmt3, and the two maintenance methyltransferases Dnmtla and Dnmtlb, which are involved in CpG methylation (Wang et al. 2006). Because other insects such as *Drosophila melanogaster, Anopheles gambiae*, and *Bombyx mori* do not have fully functional machinery for CpG methylation, *A. mellifera* is expected to be an important model for studying epigenetics in insects (Schaefer and Lyko 2007). Interestingly, it has been reported that the knockdown of *Dnmt3* by RNAi leads to the alteration of caste differentiation so that larvae fed on an artificial worker jelly develop into queens or queen-like adults, thereby suggesting that DNA methylation mediates caste differentiation (Kucharski et al. 2008).

Hexamerins are a family of major storage proteins in insects (Telfer and Kunkel 1991). In holometabolous insects, hexamerins are usually synthesized in fat bodies and secreted in the serum at the larval stage. They are collected back into fat bodies just before metamorphosis under the regulation of ecdysteroids, and utilized as a source of amino acids during metamorphosis (Ueno et al. 1983; Telfer and Kunkel 1991; Burmester et al. 1995; Burmester and Scheller 1995). Furthermore, hexamerins appear to play an important role in caste differentiation in social insects such as *Polistes* wasps (Hunt et al. 2007) and the termite *Reticulitermes flavipes* in which the simultaneous suppression of hexamerins 1 and 2 promotes differentiation toward the caste of soldiers (Zhou et al. 2006).

*A. mellifera* has four hexamerins: hexamerins 70a, 70b, 70c, and 110, of which hexamerin 70a is expressed in both larvae and adults, and high levels of the other three are expressed at the larval stage and drastically decrease thereafter (Cunha et al. 2005; Bitondi et al. 2006). In addition, hexamerin 110 (Hex110) has been implicated in ovary development. Workers are basically sterile, but they can occasionally lay unfertilized eggs, particularly when the queen is absent from their colony. It was reported that Hex110 expression was elevated in workers that developed ovaries in the absence of the queen (Bitondi et al. 2006). This observation in honeybee workers, together with that of hexamerins in other social insects, implies that Hex110 may also be involved in caste differentiation in honeybees.

In this study, cytosine methylation in Hex110 was analyzed to clarify the methylation pattern of A. mellifera genes, and the possibility of epigenetic regulation of Hex110 expression was examined.

## Materials and Methods

### Insect materials and nucleic acid isolation

The European honeybee, *A. mellifera,* was purchased from a local supplier (Nonogaki Apiary in Aichi, Japan) and maintained in an apiary of Tamagawa University in Machida, Japan. The queens were reared by transferring the 1st instar larvae from worker cells to artificial plastic queen cell cups. RNA and DNA were extracted from the whole tissues of the last instar larvae and the fat bodies of adults younger than 24 h following emergence. Because isolating fat bodies was not feasible, the entire digestive system was removed from the abdomen and the resulting abdominal integument was used as the fat body sample (Bitondi et al. 2006). RNA was isolated using ISOGEN (Nippon Gene, www.nippongene.com) following the manufacturer's instructions. DNA was extracted by proteinase K digestion and phenol extraction followed by ethanol precipitation.

### Determination of the full-length sequence of the *Hex110* transcript

Rapid amplification of cDNA ends (RACE) was performed using RNA extracted from a worker larva and the ExactStart Eukaryotic mRNA 5′- and 3′-RACE Kit (Epicentre Biotechnologies, www.epicentre.com/main.asp). An adaptor oligoribonucleotide (5′ adaptor) was ligated to the 5′ end of the RNA, and cDNA was synthesized using an oligo(dT) primer that contained another adaptor sequence (3′ adaptor). The 5′ region of *Hex110* was amplified by PCR using a 5′ adaptor primer, 5′-TCATACACATACGATTTAGGTGACACTATAGAGCGGCCGCCTGCAGGAAA-3′, and a gene-specific primer, 5′-GGACGGCCTGCGAATTCAAGTCCATTGAGACCGCG-3′.

The PCR products were cloned into the pCR2.1-TOPO vector (Invitrogen, www.invitrogen.com) and were sequenced using the BigDye Terminator v3.1 Cycle Sequencing Kit (Applied Biosystems, www.appliedbiosystems.com) and an ABI PRISM 3100 genetic analyzer (Applied Biosystems). The full-length cDNA was amplified with a 3′-adaptor primer, 5′-TAGACTTAGAAATTAATACGACTCACTATAGGCGCGCCACCG-3′, and a gene-specific primer, 5′-ATCGCATCCCATCATTGAATTTCGC-3′, which were designed to hybridize with the 5′ end of *Hex110*. The PCR product was cloned and sequenced as described above.

### Northern blot analysis

RNA was electrophoresed on a 1.5% formaldehyde-containing agarose gel and transferred to a GeneScreen Plus Hybridization Transfer Membrane (PerkinElmer, www.perkinelmer.com). To prepare a specific probe for *Hex110,* a 341-bp fragment corresponding to the 2nd exon was amplified by PCR from genomic DNA using the primers 5′-CTGACCAGGATCTCCTTAAC-3′ and 5′-CTTAAGAAATTGTCCTTCATTAAC-3′, and the fragment was cloned into the pCR2.1-TOPO vector. The *Hex110* fragment was amplified once more from the cloned plasmid with the same primer set, and the resulting product was purified using the MinElute PCR Purification Kit (Qiagen, www.qiagen.com). Probe labeling with alkaline phosphatase, hybridization, and signal detection were performed using the AlkPhos Direct (GE Healthcare, www.gehealthcare.com) and a VersaDoc Imaging System (Bio-Rad, www.bio-rad.com) according to the manufacturers' instructions.

### Semiquantitative reverse transcription (RT)-PCR

First-strand cDNA was synthesized from 4.6 µ g of total RNA by SuperScript III reverse transcriptase (Invitrogen) using an oligo(dT)12–18 primer. A 181-bp fragment of *Hex110* was PCR-amplified for 25, 30, or 35 cycles using the primers 5′GAACTTGATCAATTTATCC-3′ and 5′-AACTGAAGATTTGATGTG-3′. As a control, a 181-bp fragment of *β-actin* was amplified with the primers 5′-AGGAATGGAAGCTTGCGGTA-3′ and 5′-AATTTTCATGGTGGATGGTGC-3′.

**Figure 1.**
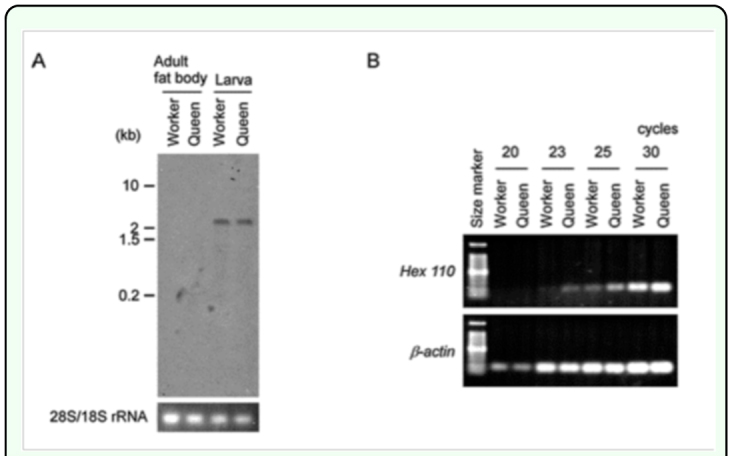
Expression of *Hex110* in *Apis mellifera.* (A) Northern blot analysis of *Hex110* in the fat bodies of adults and in the whole tissues of the last instar larvae. Bands of rRNA stained with ethidium bromide are shown in the lower panel as a loading control. (B) Semiquantitative RT-PCR of *Hex110* in the adult fat bodies. High quality figures are available online.

### Bisulfite sequencing

DNA extracted from individual *A. mellifera* was either single-digested with *Bam*HI or double-digested with *Bam*HI *and Ase*I, and subjected to bisulfite conversion using the Epitect Bisulfite Kit (Qiagen). The double-digested DNAs were used to analyze subregions 6 and 7 (Table 1). The other regions were analyzed using the single-digested DNAs. The target regions were amplified by nested PCR using the primer sets listed in Table 1. The resulting DNA fragments were cloned into the pCR2.1-TOPO vector (Invitrogen), and 8 or more clones were sequenced per amplicon.

## Results

### Cloning and expression analyses of the *Hex110* transcript

The full-length sequence of the *Hex110* transcript was identified by 5′- and 3′-RACE. The 3190-nt sequence determined (accession no. AB549723) consisted of a 26-nt 5′ untranslated region (UTR), 3024-nt coding sequence (cds), and 140-nt 3′ UTR. The cds determined was 9 nt shorter than the cds predicted from the *A. mellifera* genome sequence in the NCBI database (NM_001101023). Except for this deletion, the determined cds was 99.9% identical to the predicted sequence.

The Northern blot analysis demonstrated that the expression of *Hex110* was much higher during the larval stage than at the adult stage regardless of the caste (Figure 1A). Although the expression could not be detected from adult fat bodies in the Northern blot analysis, semiquantitative RT-PCR results suggested that *Hex110* was expressed in the adult fat bodies and that the expression level was higher in the queen than in the worker (Figure 1B), which is consistent with the view that *Hex110* is involved in ovary development (Bitondi et al. 2006).

### Methylation pattern in the *A. mellifera Hex110* gene

**Figure 2.**
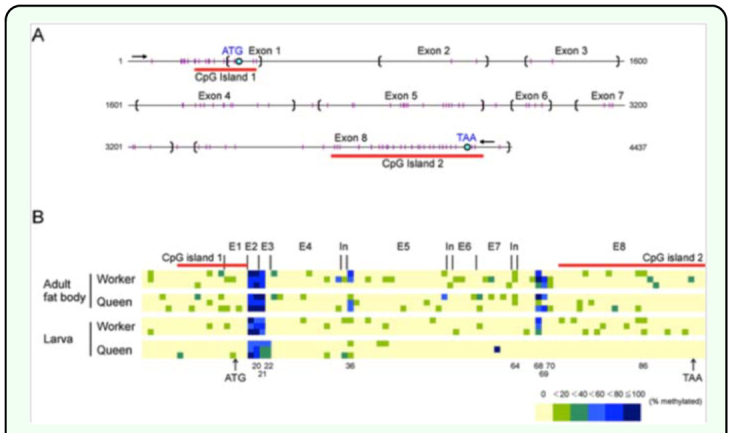
Structure and methylation pattern of the *Apis mellifera Hex110* gene. (A) Genetic structure of *Hex110.* The analyzed region is indicated by a set of arrows. The parentheses represent the exonintron boundaries. The small vertical lines indicate individual CpG sites. The CpG islands were identified with a CpG Island Searcher algorithm (http://www.uscnorris.com/cpgislands2/cpg.aspx) with the following criteria: a GC content of at least 50%, a ratio of observed-to-expected CpG frequency of at least 0.6, a region length of 200 bp, and a gap between adjacent islands of at least 100 bp. (B) CpG methylation map of *Hex110.* All CpG sites within the analyzed region are shown in three replicates for each group. E, exon. In, intron. High quality figures are available online.

The methylation profile was analyzed for a 4242-bp sequence of the *Hex110* gene including the 244-bp region upstream from the transcriptional start site (Table 2). Genomic DNA was extracted from whole larval tissues and the adult fat bodies of workers and queens with three biological replicates for each group. The examined region contained 96 CpG sites (Figure 2A) of which cytosine methylation was detected in methylation profiles were almost the same among the three replicates in each group with a few minor differences (Figure 2B). Furthermore, the methylation patterns were similar between the groups. Overall, the methylation level was low throughout the *Hex110* gene. There were several highly methylated CpG sites that were limited to a few exon regions, such as exon 2, exon 3, and the N-terminal region of exon 8. The methylation level in the upstream region of the transcriptional start site was low.

Methylation levels were higher in the adults than in the larvae regardless of the caste: the average methylation rates were 3.3% in worker larvae, 5.5% in worker adults, 3.0% in queen larvae, and 5.3% in queen adults. There were 6 CpG sites (sites 20, 21, 36, 64, 68, and 69; Figure 1B) where the methylation levels were significantly higher in the adults than in the larvae (Mann-Whitney *U*-test, *P* < 0.05, Table 2), while no larval-biased methylation site was found. The average methylation rates were nearly identical between workers and queens in the larval and adult stages. In the larval stage, however, 4 CpG sites (21, 22, 68, and 87) were significantly different between castes (Mann-Whitney *U*-test, *P* < 0.05). In addition, almost no methylation was observed in the 3′-half region exclusively in the queen larvae.

A computational analysis of the *Hex110* sequence identified two CpG islands: one in the 5′ region containing the transcription start site and translation start codon, and the other in the 3′ region containing the stop codon (Figure 2A). The methylation levels of the CpG island in the 5′ region were low with no remarkable differences among the four groups (Figure 2B, Table 3). The CpG island in the 3′ region overlapped with the region where no methylation was detected in the queen larvae.

## Discussion

This study analyzed cytosine methylation of the *Hex110* gene with a single-base resolution by bisulfite sequencing. DNA methylation in *A. mellifera* has been examined for several genes, but these analyses were limited to partial regions of gene fragments (Wang et al. 2006; Kucharski et al. 2008). To our knowledge, this is the first report on the methylation pattern of an *A. mellifera* gene encompassing the 5′-upstream and full-length cds. Overall, the methylation level of the *Hex110* gene was low, while several CpG sites within the exons were highly, but not completely, methylated. The methylation pattern observed in the *Hex110* gene is consistent with the previously reported feature of the methylation of *A. mellifera* genes, suggesting that partial and moderate methylation is a general characteristic of *A. mellifera* DNA. Such a methylation landscape appears to be quite different from that of other animals. The DNA of mammals is highly methylated throughout the genome with the exception of short unmethylated CpG islands in the promoter regions (Ball et al. 2009; Lister et al. 2009). In invertebrates, DNA methylation has been analyzed in detail in the tunicate *Ciona intestinalis* whose genome shows a clear mosaic pattern consisting of relatively long, almost completely methylated and nonmethylated regions (Suzuki et al. 2007). The methylated regions are restricted to gene bodies, while the non-methylated regions are found in both gene bodies and in the intergenic regions in *C. intestinalis.*

The *Hex110* gene has two CpG islands, one of which is located in the 5′ region, which also contains the transcriptional start site. In mammals, methylation of the CpG island in a promoter region typically correlates with the transcriptional silencing of imprinted genes (Stöger et al. 1993) and with genes on the inactive X chromosome in females (Mohandas et al. 1981). The CpG island found in the 5′ region of *Hex110* was methylated only at low levels in the four groups examined, revealing no indication of their function. Judging from its location, however, it is conceivable that the CpG island may have some roles in *Hex110* transcriptional regulation.

Because methylated cytosines are hypermutable due to spontaneous deamination, which causes a gradual depletion of CpG dinucleotides from methylated DNA regions on an evolutionary time scale, the frequency of CpG dinucleotides is a robust measure of the level of DNA methylation (Bird 1980; Elango et al. 2009). A computational analysis of CpG contents in the *A. mellifera* genome indicated that approximately 35% of the genes are expected to be methylated, and the microarray profiling of several tissues suggested that most of the genes predicted to be methylated are associated with housekeeping roles (Foret et al. 2009). In this context, the *Hex110* gene may be a rear gene that is methylated and exhibits temporal- and tissue-selective expression. *Hex110* expression was high in larvae, but the methylation levels were lower in the larvae than in the adult fat bodies. Although there is insufficient information to discuss the relationship between transcriptional activity and gene body methylation in *A. mellifera,* it is possible that *Hex110* expression is epigenetically regulated. It was also demonstrated that the *Hex110* gene was methylated in a caste-selective manner at the larval stage: there were several CpG sites highly methylated either in workers or queens, and the 3′ region containing a predicted CpG island was not methylated exclusively in queens, which was repeatedly observed in the three biological replicates derived from three colonies. This difference might be due to varying tissue compositions between the queen and worker larvae. However, we think that it is more likely that the differences reflect caste-specific epigenetic regulation because the larval body is relatively simple in structure and the tissue composition is more or less similar between the castes.

Studies have suggested that gene body methylation is associated with a variety of epigenetic phenomenon (reviewed in Suzuki and Bird 2008). In the flowering plant *Arabidopsis thaliana,* heavily methylated regions include transcriptionally inactive heterochromatin and transposons, in line with the classical view that DNA methylation contributes to gene silencing (Zhang et al. 2006; Zilberman et al. 2007). However, CpG methylation in *A. thaliana* also covers the transcribed regions of many genes, especially those of housekeeping genes. The preferential methylation of housekeeping genes is also observed in *C. intestinalis* (Suzuki et al. 2007). A recent high-throughput analysis of the human genome revealed that high methylation levels in the gene body tend to correlate with higher expression of a protein-coding gene (Lister et al. 2009). The accumulating evidence will require comparative biological analysis in the future to reassess the functions and biological significance of DNA methylation. The *A. mellifera* is a new model that provides the opportunity to study the epigenetic regulation of phenotypic plasticity in social insects. *Hex110* is a luxury gene that exhibits developmental stage-, tissue-, and caste-selective expression. We expect that further detailed analyses on the methylation of this gene will provide insight into the functions of DNA methylation in *A. mellifera.*

**Table 1.**
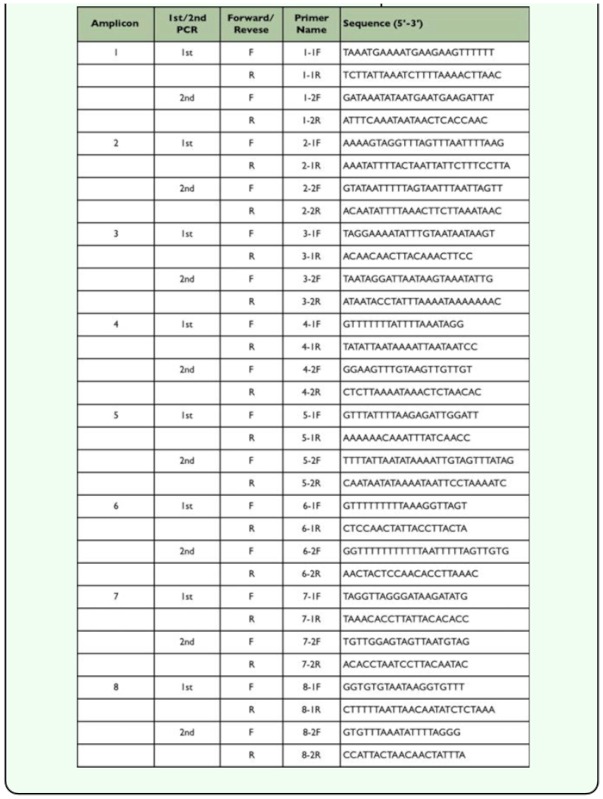
Primers used in the bisulfite sequence of *Hex110.*

**Table 2.**
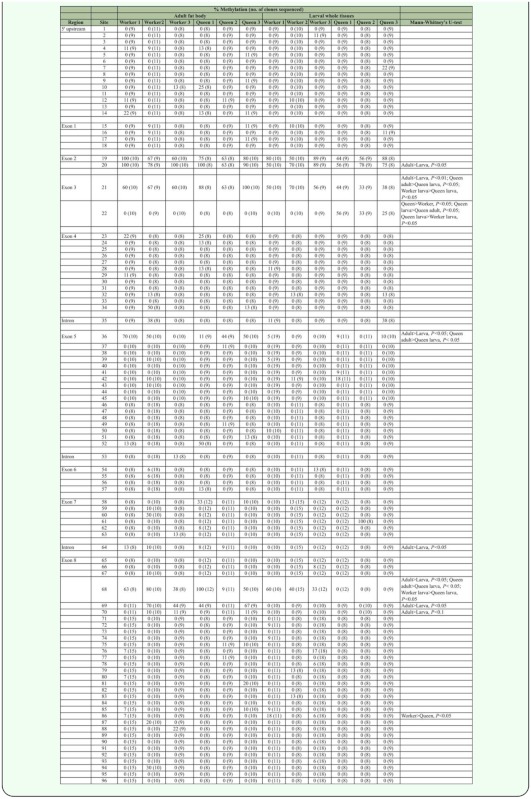
Methylation rate at each CpG site of *Hex110 of Apis mellifera.*

**Table 3.**
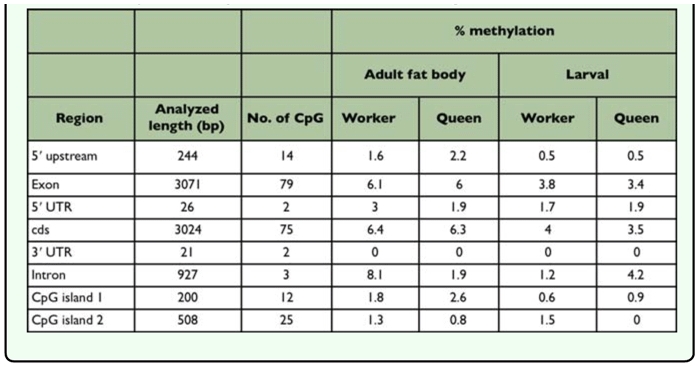
CpG methylation in the *Hex110* gene of *A. mellifera.*

## Abbreviations

**cds**,: coding sequence;
**Hex 110**,: hexamerin 110;
***RACE***,: Rapid amplification of cDNA ends;
**RT-PCR**,: reverse transcription-PCR;
**UTR**,: untranslated region
